# “Doing Good” in U.S. Cancer Genomics? Valuation practices across the boundaries of research and care in rural community oncology

**DOI:** 10.1080/14636778.2022.2091532

**Published:** 2022-07-12

**Authors:** Katherine Weatherford Darling, Michael Kohut, Susan Leeds, Eric C. Anderson, Paul K.J. Han

**Affiliations:** a Social Science Program, University of Maine at Augusta, Bangor, ME, USA;; b Center for Interdisciplinary Public & Health Research, MaineHealth Institute for Research, Portland, ME, USA;; c Tufts University School of Medicine, Boston, MA, USA;; d National Cancer Institute, National Institute of Health, Bethesda, MD, USA

**Keywords:** genomic tumor boards, cancer genomics, precision oncology, rural cancer care delivery

## Abstract

Genomic Tumour Testing (GTT) is an emerging site of “experimental care” in oncology [Cambrosio, Alberto, Peter Keating, Etienne Vignola-Gagné, Sylvain Besle, and Pascale Bourret. 2018a. “Extending Experimentation: Oncology’s Fading Boundary Bbetween Research and Care.” *New Genetics and Society* 37 (3): 207–226. doi: 10.1080/14636778.2018.1487281]. Few efforts to implement GTT have reached community oncology practices or patients living in rural communities within the US. Drawing on interdisciplinary research on a state-wide cancer genomics initiative in the rural US state of Maine, this paper explores the valuation practices within community oncologist and cancer stakeholders accounts of “doing good” within genomic science and care. We contribute to STS literatures on the bio-economy by highlighting the affective dimensions of strategies for managing economic and non-economic values. Clinician and stakeholders negotiated de-economizing and capitalizing modes of doing good as they built local genomic platforms “for Maine.” These situated modes of doing good and feeling good via cancer genomics shaped how they navigated the ethical ambiguities of US biomedical markets across the boundaries of research and care.

## Introduction

Tumor somatic testing or profiling uses next-generation sequencing and other molecular biomarkers to characterize cancer tumors and make predictions about treatment responsiveness and disease prognosis (Schwartzberg *et al*. 2017; El-Deiry *et al*. 2019). Genomic Tumour Testing (GTT) has been widely implemented in oncology within genomic research initiatives based primarily at academic medical centers in large US cities but is less common in community oncology settings (Gardner *et al*. 2021). GTT has become synonymous with precision oncology and emblematic of formations of genomics that blur and re-work the boundaries between biomedical research and the delivery of clinical services (Nelson, Keating, and Cambrosio 2013; Cambrosio *et al*. 2018a). In such domains of “experimental care,” genomic and molecular diagnostics are yoked to the regulatory and experimental systems of clinical trials and create “new regimes of bio-clinical decision-making” (Nelson, Keating, and Cambrosio 2013, 417). Where previously, a molecular marker became useful through its testing in randomized clinical trials (RCTs), genomic and molecular data about cancer now become actionable by making patients eligible to participate in RCT’s for targeted and precise cancer drugs (Cambrosio *et al*. 2018a). These regimes of oncology implicitly valorize the forms of bio-clinical judgement associated with elite US academic medical centers where biomedical research and innovation are central features of the institutions’ missions.

In US-based biomedicine, however, most oncology care is delivered in community oncology practices which are historically distinct and geographically distant from academic medical centers where genomic technologies have been developed and previously evaluated (Ball *et al*. 2020; Melas *et al*. 2020). Employer-based insurance and US state and federal insurance coverage regimes do not uniformly reimburse for commercial GTT (Phillips *et al*. 2018). Furthermore, most US cancer patients live far away from most RCT enrollment sites, and most US oncologists practice in places and institutions that look and feel far removed from elite centers of biomedical innovation and the prestige of genomic medicine- they serve different patient populations, and often have more limited biomedical infrastructure and significant healthcare workforce shortages (Charlton *et al*. 2015).

While recent research highlights the implementation challenges of next-generation sequencing in clinical oncology settings, less attention has been paid to how biomedical practitioners and stakeholders think and feel about the political economies and markets these genomic practices entail on the ground (Kerr *et al*. 2019; Spector-Bagdady *et al*. 2020; Kenny *et al*. 2021; Swallow *et al*. 2020). In this paper, we address this lacuna in literature by analysing how the affective dimensions of cancer genomics play a critical but underexplored role in defining “success” across the boundaries of research and treatment within US-based community oncology. By analysing accounts of biomedical professionals located outside elite urban hubs of US academic medicine, we show how clinician and stakeholders’ feelings about biomedical markets and hierarchies integrally shaped the ways they negotiated and strategically deployed the boundaries between research and treatment in cancer genomics.

Below we examine cancer genomics from the unique vantage point of an embedded ELSI study of a cancer genomics initiative with community oncologists in the largely rural US state of Maine. Work evaluating the initiative along various metrics has been previously published (Anderson *et al*. 2021; Anderson *et al*. 2021b). Here we critically reflect on what came to count as success and for whom. We analyse cancer stakeholders and participating clinicians’ perspectives on the cancer genomics initiative, and describe how they resist and reinforce valuation practices and infrastructures in US biomedicine (Dussauge, Helgesson, and Lee 2015; Kruger and Reinhart 2017). These valuation practices include a variety of social actions and activities that create, attribute or perform various cultural and economic values (Dussauge, Helgesson, and Lee 2015; Lamont 2012). We specifically ask: What valuation practices do initiative participants and stakeholders evoke or enact to justify cancer genomics in community oncology settings? How did cancer genomics become worth doing in Maine?

In what follows, we briefly situate our work within the social science literature on valuation in biomedicine and emerging bio-economies (Dussauge, Helgesson, and Lee 2015; Birch 2017b). We build on Thompson’s (2013) concept of “good science” to analyse clinician and stakeholders’ accounts of cancer genomics in Maine with specific attention to the tensions, ambivalences, conflicts and quandaries they felt in the context of a “local” cancer genomics initiative. We then describe the initiative itself and our interdisciplinary methods. Then, turning to our discussion and findings, we describe how clinicians and stakeholders in Maine manage moral and economic values by toggling between de-economizing and capitalizing cancer genomics. They de-economized cancer genomics by critiquing market incentives and minimizing economic considerations, which enables them to “feel good” about their own individual engagement with genomics in clinical care. For many clinicians, the initiative provided a workaround to the costs of commercial tests and they constructed the project as an experiment (seemingly) outside of the market. At the same time, clinicians and stakeholders also capitalized cancer genomics by constructing the project as strategic resource and asset for “elevating” the local biomedical workforce and building new bio-clinical infrastructures that attract new biotech talent and grant funding to the region; as a result they described “feeling good” about building “local” genomic platforms (Cambrosio *et al*. 2018b). We describe how the affectively good (e.g. the “feel good” nature of the project) and different modes of “doing good” (Bogicevic *et al*. 2020) manifest the tensions, ambivalence and conflicts felt by practitioners as they sought to articulate “good” science and care within US biomedical markets and healthcare finance systems they viewed as paradoxical, contradictory and flawed.

## Doing and feeling good in cancer genomics

Below, we describe how cancer stakeholders and oncologists came to see themselves as “doing good” through their engagements with cancer genomics. We analyse the affective dimensions of the work involved in cultivating and managing these feelings in light of their critiques of prevailing US models of cancer research and treatment. We build on Thompson’s conceptualization of “good science” to understand the ways that “feeling good” – or good enough – integrally shaped biomedical practitioners’ economic and non-economic valuation practices. Our analytic contribution shows how conflicted and ambivalent feelings imbued their conception and practice of “good” science and care within prevailing models of US biomedicine. We argue that paying attention to the felt sense of moral and ethical tension among biomedical practitioners provides a critical window into how value practices and infrastructures come into conflict and how such practices might be opened up to more reflexive or collective intervention.

Critically engaging and observing the ethics integral to stem cell science in California, Charis Thompson argues that new frameworks and practices are needed to grapple with the “deeper intertwining of science and ethics” (Thompson 2013, 29).^1^ She offers “good science” as a way of asking how internal norms of scientific conduct or notions of scientific excellence might be more explicitly connected to moral or ethical questions about how society ought to be structured. Questions about “good” scientists and science are oriented towards ethical, political and moral concerns that imbue ongoing technoscientific practices: What kinds of values and politics are built-in to the design of biotechnologies? How are normative questions implicated in and through these practices? Thompson’s concept of good science opens these reflexive questions and allows her to interrogate articulations of biotechnology and conceptions of public, social and economic “goods,” including the dominant “pro-curial” ethical framework that emerged within public stem cell science in California. Inspired by Thompson, Asdal and colleagues (2021) similarly propose the “good economy” as a concept to investigate the notions of “the good” that are performed within the policy strategies of the bio-economy in Norway. This “good economy” promises not only future market potential but also qualitatively or normatively “better” life (Asdal *et al*. 2021). Similarly, Bogicevic and colleagues (2020) study of experimental cancer care in Danish healthcare identified two distinct “modes of doing good” via germline and somatic genomic information. Through these patterns and ideals, what counts as good in one mode might be evaluated differently in another mode or context. Taken together, these scholars interrogate how social, ethical, economic and bio-clinical notions of the good are wedded within contemporary technoscientific practices and broader visions of better or more desirable futures. Here, we extend these analyses to examine interrelated and contested accounts of “doing good” in and through cancer genomics in community oncology practices located in Maine: What does “good” genomic science and “good” cancer care look and feel like to participants in a cancer genomics program in a rural US state, now and in the future?

These questions put us in conversation with interdisciplinary scholars interested in the ways that values are manifest and transformed through practices which are simultaneously moral and ethical and as well as biomedical, political and economic (Cooper 2008; Sunder Rajan 2012; Dussauge, Helgesson, and Lee 2015; Birch 2017b; 2017a; Datta Burton *et al*. 2021). Valuations and evaluations are judgements that assign worth and worthiness within a specific hierarchical order, which may or may not be readily quantifiable or monetized (Lamont 2012; Kruger and Reinhart 2017). The relational and interactional dynamics of evaluation and valuation are intimately linked and deeply embedded in specific communities (Lamont 2012), and bound to value infrastructures or systems by which orders of worth are negotiated (Kruger and Reinhart 2017). We see valuation as inextricably linked to “judgments about what is good, right, moral, beneficial, harmful risky or dangerous within a particular context or community of practice” (Weatherford Darling *et al*. 2015, 4). The political economies of biomedicine are thus tightly – and tensely - linked to the moral economies of clinical biomedicine, genomics and bioinformatics (Daston 1995; Ackerman *et al*. 2016). These dynamics involve power struggles over questions “about proper criteria and about who is a legitimate judge” (Lamont 2012, 204). Where health and more and better life operate as strong moral and market imperatives (A. E. Clarke *et al*. 2010), biomedical practitioners are building “concrete answers to the ethical question of “how to act”” (Weatherford Darling *et al*. 2015, 4) at the same time that they are constituting what it means to be a “good” scientist or clinician (Ackerman *et al*. 2016), and growing biomedical markets (Dumit 2012; Kaufman 2015). Following Dussauge and colleagues’ (2015) attention to how values are enacted, cultivated and managed in sociotechnical assemblages, we resist defining values in terms of predefined concepts like “clinical utility” or “research value,” and instead attend to the practices and infrastructures involved in making and enacting values: How are practitioners “moral economizing” (Dussauge, Helgesson, and Lee 2015) as they engage with (or seek to avoid) biomedical markets? Taking this “values-in-the-making” approach (Dussauge, Helgesson, and Lee 2015), we contribute to the understanding of emerging genomic practices in community oncology settings by highlighting the work involved in cultivating these “good” actions and feelings among cancer stakeholders and oncologists.

By integrating affective dimensions of contemporary US cancer cultures and economies with Dussauge *et al*. “valuography” (2015), our analysis contributes to the understanding of value practices as negotiated via both structures of knowledge *and feelings* within cancer genomics and clinical trials specifically (Jain [1967] 2013; Kaufman 2015; Swallow *et al*. 2020). Dussauge and colleagues argue that “value/s refers to orderings that are simultaneously of meaning/knowledge and emotional” (Dussauge, Helgesson, and Lee 2015, 9). Despite building concern for burnout, moral injury and the emotional socialization of physicians (Underman and Hirshfield 2016; Vinson and Underman 2020), the understandings of valuation practices that operate on emotional and affective levels remains understudied.

Current literature underlines how genomics’ promissory rhetoric is coupled with distinctive affective states, including anticipation, hope and worry, or feelings oriented to the future (Fortun 2008; Adams, Murphy, and Clarke 2009). For example, Kenny and colleagues observed that Australian clinicians and patients encountering cancer genomics felt the “problematic pull of survival” regardless of affective or material costs (Kenny *et al*. 2021, 2185). Feelings of hope and anticipation then drive “participation in precision innovation” in the present while reinforcing “potential efficacy” and “hope for future cures” (Kenny *et al*. 2021, 2187). Swallow and colleagues recently highlighted these affective dynamics of genomic cancer trials, arguing that the “successful management of advanced cancer patients’ expectations and emotions” by care-workers were integral to defining the success of a large UK based trial (Swallow *et al*. 2020, 2). Kerr and colleagues (2019) have similarly shown how professional uncertainty work shaped the ways clinicians, geneticists and pathologists managed expectations in implementing genomics amidst national and local anxieties in the resource-strapped UK health system.

Within US cancer cultures and economies, Jain ([1967] 2013) and Kaufman (2015) have each examined how the ascendancy of clinical trials in oncology and more widely within biomedicine not only industrialized biomedical knowledge production but also reshaped emotional and ethical dynamics of cancer treatment decision-making. Dr. Jain’s evocative ethnography documents deeply painful experiences of “not knowing” and endless “events that have no metric of comprehension” that cancer treatment entails ([1967] 2013, 217). Dr. James and colleagues show how these means of “living in prognosis” intersect with raced and classed forms of precarity, involving both calculable risks and a felt sense of safety, normalcy, and control (James 2016; Joyce, James, and Jeske 2020). These scholars critically examine the bioclinical work involved in managing cancer patients’ expectations, desires, hopes, sadness and mourning in precision oncology. In contrast, our data speak to the ways clinicians and stakeholders navigated their own ambiguous feelings about the ethics of US biomedical markets. Our analytic contribution therefore emphasizes the “emotional valence” of value practices within “competing orders of desirabilities” operating within cancer genomics and rural healthcare systems (Dussauge, Helgesson, and Lee 2015, 9).

These tensions and ambiguities felt by clinicians and stakeholders were negotiated through two modes of “doing good” via cancer genomics: de-economizing and capitalizing. At the same time that clinicians and stakeholders framed the initiative as a non-commercial space, they saw cancer genomics as a platform for regional economic development and a strategy for strengthening the biomedical workforce. Minimizing economic viability, expanding “access” and building local community allowed cancer stakeholders and oncologists to feel good about doing cancer genomics while simultaneously critiquing insurers, pharmaceutical marketers and contradictions within the broader value infrastructures of US biomedicine.

## Research context: The Maine Cancer Genomics Initiative

Rural US states, such as Maine, provide a particularly unique empirical site for understanding emerging genomic platforms (Cambrosio *et al*. 2018b). Rural communities in the US have overall worse health outcomes, shorter life expectancy, and higher prevalence of chronic disease, and receive less medical care than urban counterparts (Ziller 2015). Cancer incidence and mortality are also higher in rural vs. non-rural areas, making research on rural cancer control an emerging priority for the National Cancer Institute (Blake *et al*. 2017). US cancer leaders argue that poor health outcomes for rural cancer are driven by lack of providers, low rates of insurance coverage and limited access to emerging cancer technologies and clinical trials (Yabroff *et al*. 2020; Levit *et al*. 2020). These technologies are typically developed by universities and private technology companies and initially implemented by urban academic medical centers, before being disseminated more broadly into community practices and rural settings.

In 2016, the Maine Cancer Genomics Initiative (MCGI), an ambitious state-wide program aimed at disseminating cancer genomics in community oncology practice settings, was created by a 5-year, multi-million dollar philanthropic grant. The MCGI was organized and led by a non-profit research and education institute (referred to hereafter as the “Laboratory”) that included a regional center in Maine. The initiative began with the ambitious goal of providing tumor genomic testing for 1800 Maine cancer patients over five years. Importantly, the GTTs were offered free of charge without any medical billing. The MCGI became one of the few efforts to implement GTT in community-based oncology practices and rural communities in the US. Previous initiatives have typically been located within large, urban, academic medical centers in which research and clinical services are co-located (Cobain *et al*. 2021; Nadauld *et al*. 2018; Zehir *et al*. 2017).

Rather than being affiliated with a single medical center or health system, the MCGI was open to all community oncology practices in the state. Clinicians were also invited to participate in virtual Genomic Tumour Boards (GTBs) convened to discuss and interpret genomic results with MCGI staff, Steering Committee Members and expert consultants based in elite US academic medical centers. Once oncology clinicians joined the MCGI, they were able to offer free tumor sequencing to their patients as part of their clinical care. Often the genomic sequencing protocols utilized existing tissue samples from biopsies or surgical treatments. Clinicians enrolled patients with a variety of malignancies and at different stages of their cancer journey. In addition to receiving genomic sequencing results, patients were invited to join a research study associated with the MCGI that included participating in surveys and a registry database (for more details about the patient population and research study, see Anderson *et al*. 2021).

## Methods: embedded interdisciplinary ethical legal & social implications (ELSI) research

The current embedded ELSI project was part of a larger mixed-methods evaluation of the MCGI, including longitudinal surveys and in-depth interviews with participating clinicians. Here we focus on the analysis of the qualitative in-depth interviews, with findings from the surveys reported elsewhere (Anderson *et al*. 2021c; Anderson *et al*. 2021b; Fenton *et al*. 2021; Han *et al*. 2021). We used a modified grounded theory approach to develop and refine the focus of the qualitative data analysis. A thematic analysis of clinician interview transcripts identified key relevant areas, including the value of GTT for clinicians, extant patients and future patients, as well as perceptions of the boundaries of research and care in oncology. To follow-up on these themes, one team member conducted additional in-depth interviews with Cancer Stakeholders, including biomedical experts and practitioners involved in the projects and public health and health systems leadership in Maine. These interviews captured perspectives and experiences with implementing GTT and precision oncology from the vantage points of diverse institutional positions and professional roles.

### MCGI Clinician Interviews:

We conducted in-depth interviews with 19 oncologists and one nurse practitioner participating in MCGI. Clinicians practiced in a variety of healthcare settings, including community practices and larger health systems across the state.

### Cancer Stakeholder Interviews:

To explore further themes identified in Clinician interviews, Weatherford Darling developed a new interview guide and conducted interviews with Cancer Stakeholders in Maine. Weatherford Darling conducted 14 interviews with a purposive sample of cancer stakeholders in Maine, including biomedical and genetic scientists, and cancer leaders in public health, philanthropy and health systems. To protect participants’ confidentiality, all these participants are de-identified as “Cancer Stakeholders” in the discussion of our findings.

Table 1 details the composition of study participants.

### Data Analysis:

Clinician interviews were professionally transcribed and uploaded to the software program MAXQDA^™^ to facilitate analysis. The clinician interview transcripts were coded using thematic analysis with memos discussed by the research team (V. Clarke *et al*. 2019). Authors agreed that key themes related to costs, economics and value within cancer genomics should be further investigated. The analysis team returned to the transcripts and identified additional excerpts related to these themes, including any utterances that potentially undermined those themes (i.e. negative cases), and developed a thematic map to explore relationships among concepts. Stakeholder interviews were auto-transcribed by Zoom and edited transcripts were uploaded to NVivo. Weatherford Darling coded the transcripts using the principles of grounded theory and situational analysis, with specific attention to conceptualizing the processes of valuation and the ways that multiple discursive positions were articulated by actors (A. E. Clarke 2005; Charmaz 2014). This analytic strategy drew focus on the ambivalences and tensions within and discourses and practices across actors’ perspectives and accounts, as well as the role of standards and classification systems.

### Ethical Approval:

The protocol for stakeholder interviews was reviewed and determined to be IRB exempted by the University of Maine at Augusta IRB. The protocol for in-depth interviews with MCGI Clinicians was reviewed and approved by the Western IRB.

## Discussion & findings

The primary goals of the MCGI as a research initiative were to enrol patients, conduct tumor sequencing, and collect data on patient and physician outcomes, including data on the number and outcomes of patients recommended for targeted anti-cancer treatments or referred for participation in clinical trials. In this paper, we do not answer questions as to whether or not MCGI was successful according to any of these measures. Instead, we track interlocutor’s accounts of “doing good” through their engagement with cancer genomics, analysing where and how they were articulating specific notions of “good” science and care within and through MCGI. By examining how clinician and stakeholders anchored these valuation practices, we identified two interrelated dynamics within clinicians’ and stakeholders’ valuation practices. First, *de-economizing* the cancer genomics initiative by minimizing economic considerations and removing costs to patients was critical to making clinicians and stakeholders feel like they were not simply participating in the marketing of genomics or a market-driven effort to maximize profit. By operating outside the space of commercial GTT, clinicians could justify asking patients if they wanted tumor sequencing at no upfront cost (but with uncertain and potentially unsustainable downstream consequences). Second, MCGI was seen as “doing good” by *capitalizing* cancer genomics – that is, building new assets and resources in the form of local not-for-profit biomedical research infrastructures that could attract US federal funding, attract and retain speciality trained physicians, and convince more cancer patients to stay in Maine for their treatment. Creating virtual GTBs helped clinicians feel more comfortable with genomics and more connected with elite genomic experts and academic institutions, which some described as helping them become “better physicians.” Importantly, MCGI cultivated the sense among clinicians that they were becoming more discerning about exciting “cutting edge” cancer treatment and participating in the epistemic cultures of genomics, without making them feel like they were simply being “sold” a new product.

### Doing good by de-economizing

Clinicians critiqued for-profit genomic testing companies and the “business model” of precision oncology itself, describing their frustrations in working within “upside down” biomedical systems with flawed incentives. Cancer stakeholders explicitly de-linked the value of the initiative from the potential commercial viability of a future test and strategically minimized economic considerations in the design of the project and in communications with participating clinicians. In the process, the boundaries of research and care were strategically and ambiguously recreated; implementing cancer genomics through a research protocol promised access to a potentially beneficial technology while seemingly creating an experiment outside of the current GTT market. De-economizing cancer genomics made clinicians feel more like research colleagues than customers, and allowed them to disregard or avoid the flaws of market-driven biomedicine.

#### Working in “upside down” biomedical systems

Clinicians and cancer stakeholders critiqued the pharmaceutical industry and commercial genomic testing companies as well as broader economic problems in US biomedicine. Precision oncology also reflected the “broken” US healthcare system, accentuating the flaws of a system solely “based on profits.” For example, one cancer stakeholder described genomic tumor testing as emblematic of broader problems of inappropriate test utilization in US biomedicine:

[MCGI’] got a real research goal, but I still find so often that clinicians – even beyond oncology and within oncology – don’t know what tests to order. [Clinicians] don’t order it at the right time. [We are] spending way too much money on tests, but for me it’s just as much [that they] didn’t order a test that would have been great value and … I don’t know what can be done about that.

(Cancer Stakeholder #3, 623)

Another cancer stakeholder questioned the long-term sustainability of the economic model of targeted therapies:

You know, for this model to be sustainable, at some point, you’re going to have to pay the piper for these drugs and, like, how is that all going to work? Because as a higher percentage of your population because of advancing research becomes potentially a candidate for a precision therapeutic and trials are no longer being done because the therapeutics been shown to be efficacious. Right, will the drug costs come down sufficiently or are they gonna stay very high and, like, how is that all those lines of cost and benefit going to intersect?

(Cancer Stakeholder #16, 458)

The actionable outcome of tumor sequencing was eligibility for a clinical trial or “off-label” use of a drug, but as this stakeholder wondered, it was unclear whether and how these targeted therapies would be affordable or widely available once they were no longer considered experimental. As one clinician explained, the frustrations of insurance hurdles was “just infuriating”:

And then the other thing, I’m sorry, I get frustrated with this right now because I’m running into this issue. … if we do find some potential treatments, especially when there’s nothing else to give to the patient, I’m having a heck of a time trying to get drugs off label and approved [by patients’ insurance]. We have to go through making [patients] insurance will deny it, and after the insurance denies it, sending it to the company and —, I mean, it just the amount of paperwork, the amount of steps, it’s just infuriating.

(MCGI Clinician #4, 24)

While MCGI provided support with requests to companies to donate drugs when they were not covered by patients’ insurance, this clinician was pessimistic these administrative hurdles could be overcome in the long-run. They said:

I’m going to be very pessimistic. The way health care is going, I don’t think we’re going to be able to overcome it. It is driven by money. Insurances are for profit. … these drugs are extremely expensive. They [insurers] don’t want to pay for it, especially if there’s not much data out there, which I can understand. And because our whole society, including health care is based on profits, I don’t see this changing at any point. … I mean, I think this comes into the overall healthcare issue. Right? Forget genomic testing. Go look at cancer care, or you look at care in general. It’s just way too expensive.

(MCGI Clinician #4)

The problems of profit-based healthcare and expensive drugs were seen as endemic to US biomedicine. Another cancer stakeholder echoed these systemic critiques and laid out what they saw as an “upside down model” in biomedicine that overly favors intensive treatment over quality of life:

… we’re not doing a good job of informed decision making … [not] good stewards of our resources, necessarily … I don’t tend to blame anybody … our culture was everyone [in medicine] gets paid for doing things, that was an upside down model. Our outcomes should be based upon quality of life, life expectancy and mitigating pain and suffering.

(Cancer Stakeholder #15, 187)

As this stakeholder explains the “culture of biomedicine” prioritizes services that are reimbursed, and those reimbursement and coverage decisions, at least in the Medicare population, have become central the “ethics of managing life” by determining what kinds of life-extending therapies become standard for older people (Kaufman 2015). In critiquing reimbursement practices and priorities as valuing the “wrong” things (e.g. technology or profits over patient care), clinicians and stakeholders expressed frustration and resignation. No one in particular is to “blame” (Stakeholder #15), and yet insurance reimbursement, specifically Medicare reimbursement policies drive the forms of evidence that make tests and treatments “standard” and “necessary” for older adults in the US (Kaufman 2015).

Each of the above examples show the ways clinicians and cancer stakeholders critiqued systemic issues with the incentives built-in to US biomedical models of financing healthcare treatments. They critiqued the criteria by which insurers paid or denied claims and systems for paying for cancer treatments. However, these critiques focussed on the off-label or experimental therapies that genomic sequencing promised rather than the sustainability of genomic sequencing supported by a time-limited philanthropic grant. In light of ethical unease and political critiques, “doing good” through cancer genomics required working around what clinician and stakeholders’ described as the flaws inherent to US biomedical markets.

#### Creating an experiment “outside” the GTT market

The framing of the initiative as a “non-commercial” research project was one important strategy for building trust with community-based oncologists. Recalling early resistance to the project, one cancer stakeholder summarized clinicians’ perspectives this way: “Well, the doctors would think, why should [clinicians] order your test you want to make so that one day you’re profitable?” (Cancer Stakeholder #13). They further explained:

… [A doctor would say] “we don’t need [the Laboratory]. I have my own testing done. I understand the results. [The Laboratory] has no idea what they’re doing. I don’t want to spend time talking to them.” … Yeah, that guy got pushed out …

(Cancer Stakeholder #13)

While these concerns dissipated over time, clinicians expressed concern about getting “honest data,” citing the Laboratory’s potential “proprietary interest”:

I hope they’re gonna collect the data on how many people got successfully treated. … They [the Laboratory] have a proprietary interest probably in wanting to eventually market this and I hope that we’re going to have honest data that it’s going to look at it. And if the data comes out that not many patients were helped, and they’re [looking at] toxicity, you know, looking at toxicity. I hope that data … will be eventually presented.

(MCGI Clinician #6, 99)

Throughout the project, initiative leaders had to address concerns that MCGI was “just trying to do this sort of as a commercial activity” (Cancer Stakeholder #11). Creating a study protocol with clear research goals helped assuage such concerns about potential profit motives, while also releasing the initiative itself from market pressures and regulatory hurdles that some stakeholders perceived as insurmountable. Initiative leaders made a strategic choice not to pursue investigational device status through the US Food & Drug Administration, and to instead develop an IRB-approved research protocol with the goal of “understanding the implementation hurdles” of tumor sequencing in community-based oncology practices (Cancer Stakeholder #11). Though it would have made insurance reimbursement for the test easier, filing with the FDA “locked in” test specifications that would have prevented improvement of the assay and laboratory procedures over time. As one cancer stakeholder explained, the “test itself” was subsumed under a study protocol:

[The Laboratory] decided to design the study protocol. It was sort of a complicated procedure where basically the test itself was actually not part of the study protocol … Or it was, but you could only order it for patients that were enrolled in the study. [The Laboratory] sort of created the separate clinical workflow, where the test was ordered through a clinical requisition … and then the patient had consented that all of the information that was, you know, in the test report … would all become study data. So it was sort of … a backdoor way of getting the test information … without the test actually be being [an investigational device]

(Cancer Stakeholder #11, 340)

In addition to framing and implementing the cancer genomics initiative as a research study, securing philanthropic funding also removed financial profit as a primary goal in the view of some stakeholders:

And then the goals do change with the foundation funding, right? … I mean, it really stopped being a largely commercial venture and because their projections for how many of these tests they were going to sell [if it could be reimbursed] … were just completely unrealistic, even with the testing now being free they haven’t hit those numbers …

(Cancer Stakeholder #3, 473)

This focus on philanthropically supported research rather than insurance reimbursement as a goal created an experiment outside of the current GTT market. The potential effects of removing financial barriers to GTT were transformed into an open-ended question that could be answered through the initiative’s research protocols and evaluation plan. While some clinicians continued to use GTT as last resort, only after other treatment options were exhausted, the potential of “free testing” also created its own kind of experiment that might more fully assess GTT. This situation made the sequencing offered by MCGI “a different kind of test” with “a very different value than what they here first designing it for” (Cancer Stakeholder #3, 489):

…. nobody was running these tests up front … Nobody gets diagnosed and just gets their tumour profiled and goes off on whatever [treatment] … But now with this test being free a lot more patients are getting tested ….Um, so that makes it a kind of a different test, you know, lends itself, much more to research. Because now you could test that patient upfront …

(Cancer Stakeholder #3, 484)

By sidestepping insurance and medical billing, free testing made it possible to sequence tumors “upfront” as means for collecting new and different kinds of data about the molecular profiles of tumors – even among patients for whom GTT might otherwise not be considered. Examples include patients with newly diagnosed cancers with accepted first-line treatments. As this stakeholder further explained:

Because now [that it’s free] you could test that patient up front know how they reacted in the way they were treated, it might just still be conventional first line therapy … you know it’s really, what all the commercial companies have always said, You can do that and we can tell you what’s going on with a tumour … Those of us in practice, we said, “Yeah, but who’s paying?”

(Cancer Stakeholder #3, 485)

The MCGI protocol could potentially be offered for free to “anybody,” regardless of not only insurance status but cancer type or stage. In this way, “free testing” became an experimental condition of the project.

In addition to avoiding the cost or insurance hassle of commercial GTT, clinicians also emphasized communitarian ethos and “home-grown” character of the initiative as project for “the people of Maine” (MCGI Clinician #2) in describing their motivations for participating in MCGI. In other words, it was important that they didn’t feel like they were participating in a biomedical market when they interacted with MCGI. For example, some clinicians contrasted the “feel good” aspect of pursuing tumor sequencing through MCGI (MCGI Clinician #2, 78) with their encounters with pharmaceutical industry representatives and commercial testing companies. One clinician described these different feelings this way:

….it’s fascinating. It kind of keeps you, meaning me, in the game. You’ve gotta have some hope that you’re gonna have a breakthrough, right? Or you can’t keep doing this every day. So talking to the people from [Commercial GTT or Precision Medicine Companies] or from [the Laboratory] is intensely more interesting than a [pharmaceutical] drug rep who’s like peddling you … So I think it’s part of ongoing education, staying engaged, staying current, being part of a project like this. Maybe it helps us more than it helps patients right now. But that’s okay, I mean, there will be breakthroughs right?

(MCGI Clinician #13)

In this way, the “free” genomic sequencing provided via MCGI was folded into strategies clinicians used to manage patients’ hopes and expectations as well as manage their hope and optimism. The emotional labor of physicians – the work of maintaining hope while caring for dying patients and prescribing grueling treatments - was anchored in the possibility of future genomic “breakthroughs” realized through research initiatives like MCGI (James 2016; Vinson and Underman 2020). These feelings of hope and sense of community were missing from physicians’ experience of using commercial tests:

So I think that actually more of a reason to participate is just sort of a sense of medical community. That people are all sort of together. And you don’t get that sense of community when you’re working with a drug company. [The for-profit GTT Companies], do they really care about the people of Maine? Or do they care about the finance?

(MCGI Clinician #2, 84)

Clinicians reported that this feeling of being outside of the broader healthcare marketplace was reinforced by patients’ perceptions of cancer genomics and precision oncology. As one clinician observed, patients came to clinic with a “pretty picture” of precision medicine portrayed in advertising and marketing brochures. They explained that patients’ excessively hopeful view of precision medicine was influenced by commercials and advertising rather than physician-initiated discussions of genomic research, and that clinicians needed to counter this popular view and help patients achieve a more realistic, nuanced understanding of GTT:

I think it actually helps for them [patients] - not to be paternalistic - but I think when they see the results and they realize it’s difficult to explain they realize that precision medicine is a little bit more nuanced than like an ad of people walking on the beach at sunset. [For patients] it’s like, “Oh, this actually is science and I have no idea what this means and I should probably stop asking, I think.”

(MCGI Clinician #2, 66–68)

As this clinician described, the sense that MCGI represented “actual science” rather than dubious pharmaceutical marketing shaped how both clinicians and patients *felt better* about engaging with cancer genomics via MCGI. Below, we describe how the moral imperative of broadening patients’ healthcare access also reinforced clinicians’ sense of “doing good” through cancer genomics.

#### “Access” as moral anchor and counterweight

In clinicians’ and stakeholders’ accounts of “doing good” through cancer genomics, “access” served as both a moral anchor and a counterweight. For example, many clinicians described genomic tumor sequence results as academically interesting but not (yet) clinically useful to their patients. They expressed a tension between their own desire to learn about the molecular biology of cancer and their ethical ambivalence about the economic costs borne by patients:

… I want to know more about these tumours and I want to learn. But I also don’t want to be subjecting a patient to an unnecessary test or unnecessary cost if it’s not going to impact their clinical management.

(MCGI Clinician #2, 39)

Clinicians did not feel it was ethical to “pass on” the high costs of genomic sequencing to their patients, given the low yield of actionable results. They expressed a professional tension between scientific curiosity and delivering high-value clinical treatments to their patients:

….I always had apprehension about [commercial GTT’s], because they’re third party for profit enterprises and I’m passing the cost on to patients. And I didn’t feel like it was really moving research questions forward, whereas, with [the Laboratory] trial I feel like, frankly anybody could be [tested] - I would offer it to anybody because that’s the only way we’re going to figure this out

(MCGI Clinician #3, 51)

Another clinician explained they used research protocols like those offered by MCGI and academic medical centers out of state as a workaround to the expense of commercial products: “ … that was my way of going around it” (MCGI Clinician # 4, 36). “Free” testing enabled clinicians to balance the tensions between their own academic curiosity, their desire for future knowledge, and the low probability that patients would benefit directly or immediately. One clinician explained the balance this way:

Because I think it helps patients, hopefully. Maybe it’s only a small number of my patients that it helps directly. But I think down the line it will help more because it will help broadly cancer therapy, and science even more broadly. So I think it’s just kind of like a good thing to do. […] And it makes me feel good to have done that through the [Initiative], better than having done it through [for-profit company] or [for-profit company] for whatever that’s worth. So that’s the best part.

(MCGI Clinician #3)

Oncologists felt less able to justify the uncertainty surrounding tumor sequencing if the financials costs were incurred by patients directly.

In summary, de-economizing cancer genomics – that is, de-coupling MCGI’s success from the goal of creating a new marketable test and creating a space that felt removed from biomedical marketing – made it possible for clinicians and stakeholders to frame the initiative as an experiment conducted outside of the marketplace. As each of these examples show, clinicians’ own hopes for future breakthroughs as well as the feeling “of medical community” helped them see their participation in the cancer genomics initiative as a fundamental good. The MCGI allowed them to avoid not only the costs of commercial genomic tests but the negative feelings they associated with participating in “commercial” forms of biomedicine interested only in maximizing the profitability of a new technology. This felt sense of working outside of commercial space and the moral weight of access regardless of ability to pay anchored these accounts of good genomics.

### Doing good by capitalizing

Although many clinicians and stakeholders de-economized cancer genomics as a means of justifying the good of the effort, they also capitalized cancer genomics by highlighting the assets and resources created by the initiative. Clinicians and stakeholders cited the creation of three key resources in particular: the initiative’s popular GTBs, bioclinical infrastructure for both processing human tumor tissues, and an interpretable annotated database of molecular profiles to guide clinical decisions. In these ways, clinicians and stakeholders linked genomic knowledge to forms of social and cultural capital in terms of bettering their own professional judgement and networks. Importantly, these ways of capitalizing cancer genomics by creating assets in bio-economies (Birch 2016) were especially effective in engendering a mode of doing good for clinicians and stakeholders because they were not-for-profit. Rather, the GTBs and bio-clinical data infrastructures of the MCGI were seen as assets for specifically and directly enhancing the local biomedical workforce and research enterprise, while generally and indirectly promoting economic development in the state. Maintaining such “bio-clinical collectives” (Rabeharisoa *et al*. 2009) and “bio-clinical platforms” (Cambrosio, Campbell, and Bourret 2020; 2021) involved not only data infrastructures and interoperability, but also significant community-building. We extend these previous analyses of platform building by emphasizing the broad and contested range of valuation infrastructures implicated in their construction. Where Cambrosio and colleagues (2018) emphasize role of regulations in shaping the “proper” clinical uses of genomic platforms, we highlight how the negotiation of a wide range of valuation infrastructures became critical to shaping how the work of building a genomic platforms comes to be seen as “doing good.”

#### Cultivating epistemic acculturation: building a virtual Genomic Tumour Board

“Unbelievable,” “wonderful,” “fantastic,” “extremely fascinating,” “extremely interesting and educational”: Clinicians used effusive superlatives to describe the MCGI virtual Genomic Tumour Boards (GTBs). These popular forums became a site of learning and collaboration that enhanced clinicians’ epistemic cultural capital by both connecting them to elite genomic experts and making them feel more comfortable with the vocabulary, norms and practices of genomics.

The initiative cultivated clinicians’ awareness of the promise of targeted therapies and their “comfort-level” of with using molecular profiling for tumors, laying the groundwork for future research and preparing them to deliver “twenty-first century” cancer care. Clinicians felt good about developing professionally through their participation in genomic research via the project:

So this has been a wonderful opportunity for me to work and collaborate with those up at [the Laboratory] where it promotes sort of my research interests. Makes me think broader rather than just the actual clinical picture. And I think it overall makes me a better physician.

(MCGI Clinician #4, 93)

MCGI symbolized the future of biomedicine. GTBs provided the infrastructure for creating local interpretative communities around genomic sequence data and sparked an epistemic culture change among community oncologists (Knorr-Cetina and Reichmann 2015). MCGI provided direct links to precision oncology experts from academic medical centers who served as educators and translators of sequencing results. One cancer stakeholder explained how the GTBs contributed to clinician’s learning and “comfort level” with the complexity and uncertainty of genomic data, and the real-time connections to genomic experts supported this acculturation process:

I think that [the GTBs] is another thing that got the oncologists in Maine to see real value … they have access to these experts at large academic medical centres. … Like even if a patient doesn’t really end up getting targeted therapy. They’re just learning so much … You know, it’s just giving them access to something that’s much more common in academic medical centres. Now, it’s right here in their shop, you know … I mean they learn the language … [they] would never have gotten there that fast if just every now and then they ordered a [for-profit GTT company] and a printed report came back to them with data 10 pages long, and they were just staring at it going, “Oh”. They’ve got awareness now. They’ve got a comfort level. They’ve got access to specialists, they can even now make their way through one of those reports better on their own ….

(Cancer Stakeholder #3, 559).

In contrast, a cancer stakeholder explained that previously molecular tumor boards had a “very local feel” because everybody gathered in a hospital conference room at 7 AM on a Tuesday morning (Cancer Stakeholder #15, 430). Participating clinicians saw the initiative’s GTBs as educational and fundamentally unlike either hospital tumor boards or the interpretation support services provided by commercial GTT companies (Cancer Stakeholder #15).

The broad reach of clinician involvement was also a marker of MCGI’s success and a strategy for strengthening physicians’ networks. As one cancer stakeholder explained, the work of setting up the protocol and the tumor boards got “everybody to kind of start pulling in the same direction” within a usually dispersed group of community oncology providers who were ordinarily primed to compete for patients:

Weatherford Darling: You know, beyond just these 1800 [genomic tumour] tests, what were some of the impacts [of MCGI]?

Cancer Stakeholder #6: I’m glad you’re asking about this because that’s something that I should have been talking about more and [MCGI’s] really proud of it. Um, it’s pretty much at the theoretical maximum […] participation within the oncologists … the only reason I think [it’s] not at 100% I think every practice is at least involved in some way or another, which I think is unheard of. I mean, we have a pretty dispersed population throughout the state to get everybody to kind of start pulling in the same direction. It’s phenomenal. … everybody has seen value in this … … so to get them all on board is I think, to me, that alone seems to me worth the money

(Cancer Stakeholder #6, 426).

The community-building aspects of the project, including the “sense of medical community” and camaraderie it cultivated, were key motivations for clinicians to participate as well. They also critiqued GTT initiatives based at elite academic medical centers for not sharing data and results to local clinicians. For example, one clinician explained that proprietary and commercial tests had made them skeptical about using GTT:

…. a lot of people inevitably, because of where we live and practice, people go to Boston for a second opinion, and may have their own proprietary ones that they were doing. And they weren’t sharing their results. And the patients were coming back with no information and saying that they had this sophisticated testing done about precision medicine and it wasn’t doing anything for them. And I felt like I’m not going to add on a cost, and insurers weren’t necessarily paying for a lot for them …

(MCGI Clinician #3, 41–42).

The GTBs in particular convened oncologists from across the state in ways and the MCGI carefully avoided the historical divisions between competing medical groups and health systems, reflecting valuable relational connections beyond the strictly informational function of the GTBs. As these perspectives show, MCGI’s community-building and data-sharing practices represented significant departures from previous efforts to implement cancer genomics. In particular, the virtual GTBs organized by MCGI were framed as a vehicle for education, and collaborative interpretation of sequence results and reports.

Creating these new and highly networked bio-clinical collectives made oncologists “believe more” in GTT and both reflected and reinforced a shifting epistemic culture among oncologists:

I think it’s a unique way for us as a group of oncologists in the state of Maine to start to believe more. I guess that’s kind of the wrong verb, but to think about the importance of tumour testing. And I’ve talked to a lot of people, partners, for example, that have been sceptical, “Well you’ve got all these genetic information you can’t do anything with it.” Well in many cases you can, you have to just know how to interpret it and know how to understand it and so forth. And I just think it’s good for the whole state. Also that there’s nothing else that has ever gotten all the oncologists together in the state.

(MCGI Clinician #15)

Clinicians described these facilitated discussions as “one of the most valuable aspects” of MCGI (MCGI Clinician #5). The GTBs represented an opportunity to discuss patient cases and clinical trial options with scientists and cancer genomics experts from large urban academic research centers. The education, the connections to elite science, and community-building was “a total icing on the cake” of joining MCGI, one clinician said:

That’s been like game changing. [The GTB is] just amazingly valuable. I don’t have that with [for-profit GTT Companies] … I personally prefer that patient’s [go with MCGI] because I’m going to be able to talk to experts about their case. And I find that immensely helpful.

(MCGI Clinician #5)

Clinicians viewed the ongoing contact with experts provided by the GTBs as key to their own comfort with cancer genomics and valuable for their own learning, but also felt that a GTB run by a commercial, for-profit, genomic testing provider would likely not as effective, in part because of their non-local nature:

… So most of the commercial labs do offer that service (e.g. specialist interpretation by phone) … I don’t think any of the oncologists that I know have ever gone that route. I mean, because they don’t have time. … You know and like, “oh okay [this for-profit GTT Company] has a virtual tumour board, but now I have to listen to three other people’s patients” before you get to them …. [The MCGI GTB] is all their patients or their immediate colleagues and they see the value in spending the time with people they know. So it would be hard to commercialize it even though some of the commercial labs, try to. But it’s one of the great things about [the Laboratory] study. To be part of the study, you have to be part of those tumour boards. And then having been dragged to the tumour boards, they now see the value of them.

(Cancer Stakeholder #3, 603)

In addition to providing citations and research papers describing the molecular pathways and the meaning of mutations, as well as explicit “educational modules,” the GTBs created more informal learning opportunities through conversation with genomics experts “from away”:

… sometimes the physician thinks they know what the mutation means and they don’t, and the molecular biologist will set them straight or [the facilitator] will set them straight. So no, it’s more rounded out, it’s more clarified …. where the clinician thought that mutation X correlated well with treatment Y, when in fact, although that’s a mutation in a larger gene family, that that one didn’t correlate to a response to a drug that the clinician was selecting.

(MCGI Clinician #13)

Learning the “language” of genomics primed clinicians in community settings to think about the precision therapies of the future. In this priming process genomics experts went beyond just “giving information”; they strived to “make you comfortable” by answering specific questions with authority (MCGI Clinician #11). In other words, “[the] test came with all the support system” community oncologists needed (Cancer Stakeholder #13).

As described above, the network of local oncologists and the active and ongoing interpretation work of genomic experts became a critical resource created by the GTB. By linking clinicians to each other and to knowledgeable experts, MCGI built new epistemic communities that laid the groundwork for future clinical and translational research and enhanced clinician’s comfort-level with forms of genomic knowledge they perceived as elite and cutting-edge.

#### Growing a “local” genomic platform

MCGI clinicians and cancer stakeholders also capitalized cancer genomics by developing new bio-clinical data infrastructures they saw as assets for strengthening the state’s biomedical research enterprise. For some cancer stakeholders the potential of cancer genomics to simultaneously impact patient outcomes and catalyze a home-grown biopharmaceutical industry reflected a “win-win” (Cancer Stakeholder #6, 360). For others, the potential for biomedical research as an economic development strategy existed in a more complicated tension with the promoted goals of providing local access to targeted therapies and clinical trials. However, even though they expressed qualms and frustration about “health care based on profit” (MCGI Clinician #4), many clinicians and stakeholders ultimately embraced MCGI as a strategy for building a “local” genomic platform imbued with future economic promise.

The MCGI study protocol enabled patient data to be collected and shared across institutional siloes of tumor tissue registries and repositories and created a new conduit for patient bio-data. The initiative was a launch pad for developing clinical and translational research infrastructures and work flows, namely a clinical laboratory capable of processing human samples, a database of “molecular profiles” on tumors, and the interpretative infrastructures to link those data with clinical decisions. As one stakeholder explained, without these links to clinical data profiles of actual patients and the added annotation work, existing cancer registries languished as “just a freezer full of blood specimens or tumour specimens” (Cancer Stakeholder #3, 164). They said: “Nobody wants the tissue … they just need to mine molecular data along with clinical data” (Cancer Stakeholder #3, 217). The Laboratory enabled this kind of data mining. One Cancer Stakeholder explained the Laboratory’s strategic advantage compared to other genomic repositories this way:

So I would say there’s a lot of what I call [genomic] repositories out there. But they’re not, they don’t take it to the level of interpretation. Right? They’ll pull in all the variants, the layer that we wanted to add was how do you make sense of it? And how do you use that to make informed [clinical] decisions? …. What I learned … interacting with physicians, some physicians were like, “Just give me a list of mutations”. Yeah, [And some] were like “Connect the dots for me but keep it simple” and all these others were like, “show me the kitchen sink” … [the Laboratory’s approach] was to build a very modular system with snippets of content.

(Cancer Stakeholder #7, 59)

Asked how questions of revenue were negotiated within the non-profit model, they explained:

… So I would say the non-profit. Um, there’s a level like when you sell content like we’re selling, you’re selling trust. So [the Laboratory’s] history, their reputation and being a non-profit there’s an immediate sense of trust. So people have more conversations with you … you look at a return on investment or an umbrella of a non-profit where some of your ROI (return on investment) is because of the social good factor.

(Cancer Stakeholder #7, 110)

Selling bio-data required “selling trust,” while the ROI came in the form of a “social good factor” manifested in the form of research alliances or *Nature* papers. These assets, in turn, resulted in new “converts” among biomedical researchers and clinicians willing to use and financially support the database, annotation and interpretation:

And it helps build our reputation, which we then hope that converts that people to saying, “I really like [this database] I’ve been able to use it. They participate in other groups, there’s a level of trust. I want them to keep going. And I’m willing to pay to help sustain the program”.

(Cancer Stakeholder #7, 121)

That elusive “social good factor” of the Laboratory’s molecular profiling database also helped contribute to the Laboratory’s growing reputation within the arena of clinical and translational genomics. As this stakeholder implied, this reputational, curatorial and infrastructural work contributed to future grant proposals as well as other revenue that could keep the Laboratory going.

For some stakeholders, the promise of building a biobank and growing the Laboratory’s reputation as a biomedical and translational research center overshadowed the more public-facing emphasis on bringing precision therapies to Maine. For others, however, the goals of supporting economic development through biomedical research more readily merged within MCGI. One cancer stakeholder described these layered valuations as goals in tension:

It, it seemed to me that there was a major emphasis in MCGI around bio-banking and the potential longitudinal ability to mine data … discovery science. As opposed to MCGI predominantly to be of clinical benefit … potentially benefiting [patients] … to me there was a little bit of a tension there about the study and design and project design around, you know, what was the real versus stated or superficial goals of the project. You know, on the surface, it felt a lot like they were promoting it as this is going to help cancer patients but underneath it felt a lot more like this is going to help [the Laboratory] develop a really good biobank.

(Cancer Stakeholder #16, 275)

This cancer stakeholder identified a tension between the future promises of biobank development and the present benefits of delivering precision oncology to *current* cancer patients. However, for other cancer stakeholders, this “two-pronged” view of MCGI’s goals reflected more of a “win-win” than a contradiction (Cancer Stakeholder #11). They described MCGI as a strategy for simultaneously “bringing precision oncology” to Maine and building a locally grown platform for clinical and translational genomics research:

[The approach] was to basically do something for the good of Maine, right? Just for the state of Maine … you know, bring precision oncology to state … Kind of get all the practices and sort of, you know, raise up to the twenty-first century level of care

(Stakeholder #11, 73)

This stakeholder saw the potential for geographically redistributing “twenty-first century” care and simultaneously building clinical research capacity at the Laboratory, collaborating health systems, and community-based oncology practices. The “business opportunity” merged with the “service opportunity” of providing tumor sequencing for “free”:

[The Laboratory] had ventured from being a mouse, you know, mouse research and mouse production facility to be being a more clinically oriented … [becoming a] research organization and with a clinical laboratory and … You know, it’s really hard to run a clinical laboratory. If you don’t really have your own patients. And so the idea was you could basically create opportunity there. And frankly, both the business opportunity, as well as a, you know, kind of a service opportunity and research opportunity.

(Cancer Stakeholder #11, 78)

As another cancer stakeholder explained, the designation of the Laboratory as a biomedical research enterprise supported by the National Cancer Institute (NCI) was an asset to the state:

Maine is really fortunate, for state such as themselves, to have a NCI Centre with the capability of [the Laboratory]. I mean, there’s a part of their core grant renewals that’s how NCI, you know, releases funding every four or five, six years that I think that this was a part, you know, they’ve historically been known for the mouse around the world and have developed mouse models and they capitalize on that and they have a lot of tremendous science, but I think the NCI is trying to push them to be more translational. … They had the capability really develop their own in-house [clinical laboratory]. And they wanted to make that available. It was a natural extension to work with clinicians in the state of Maine. For being creative and trying to get funding. You know, the [MCGI] project has a national profile.

(Cancer Stakeholder #4, 173)

As this stakeholder describes, MCGI facilitated the Laboratory’s pivot from mouse models to a more translational research model that required and offered direct access to clinical outcomes data and human biospecimens. One stakeholder described this potential as an exciting “game changer”:

And I think what got us excited about it in the first place was yes. It’s very, it’s very exciting to us. It [GTT] is a game changer. But, but by the research, we are setting up we’re helping [the Laboratory] become a world class player even more … So it was it was it was part the excitement of the technology. Part of it was the economic development for the state.

(Cancer Stakeholder #6, 227)

In other words, building a “world class” research laboratory in Maine’s backyard had the potential to “really elevate the state” by attracting biomedical research talent and funding to the region (Cancer Stakeholder #6, 227).

In summary, the new bio-clinical workflows, infrastructures and collectives that MCGI created for future translational research were framed as a promising regional economic development strategy for both clinicians and stakeholders. Becoming acculturated in the elite science of cancer genomics elevated community oncologists’ status, while creating a clinical laboratory elevated the Laboratory’s reputation, prestige and economic viability. Cancer stakeholders used economic idioms, including language like “capitalizing,” “selling” and “business opportunity” to describe the benefits of building a local genomic platform. Clinicians and stakeholders also mobilized ephemeral factors (e.g. the “social good factor”) and amorphous quasi-economic intangibles like elevating the prestige of local biomedical institutions and practitioners. Thus, even as clinicians and stakeholders questioned the economic models of precision oncology and critiqued profit-driven biomedicine, these modes of capitalizing cancer genomics felt incalculably good for Maine.

## Conclusion

In this paper, we analysed the valuation practices at work in clinicians and cancer stakeholders’ accounts of doing good through cancer genomics in the unique context of implementing tumor sequencing in community oncology practices in the rural US state of Maine. We found that clinicians and stakeholders articulated uneasy and contested meanings of “doing good” within biomedical institutions they often viewed as flawed, unethical and socially and economically unsustainable. Feelings of excitement, frustration, tension about biomedical markets and moral quandaries in cancer care infused their value practices and shaped how they came to feel good about doing cancer genomics via the initiative. In the discussion above, we showed how they grappled with these tensions by toggling between de-economizing and capitalizing cancer genomics. They de-economized the initiative by strategically sidestepping concerns about the economic viability of a new marketable test and appealing to moral imperatives for access regardless of patients’ ability to pay. At the same time, they capitalized the initiative as a strategy for erecting a new human genomic platform and cultivating “better physicians” through knowledge and social connections associated with elite and “twenty-first century” biomedicine. Clinicians and stakeholders both critiqued and embraced market imperatives in biomedicine, capitalizing and de-economizing cancer genomics in sometimes contradictory and ambivalent ways. To conclude, we briefly reflect on the contributions of these findings to social studies of genomics.

We contribute to the understanding of emerging genomic practices and platforms by highlighting the affective valence “good science.” In particular we showed how capitalizing and de-economizing cancer genomics reflect the challenges clinicians and stakeholders felt as they encountered the impacts of broader contradictions and tensions within rural healthcare and biomedical research. There were moments of excitement, frustration, worry and ambivalence in the uneasy accounts of doing “good science” and “good care” within a broader biomedical and economic system that clinicians and stakeholders readily viewed as flawed and unsustainable. Despite their ambivalence, and through the work of initiative leaders and staff, oncologists ultimately felt *good enough* to enrol themselves in the effort because it was non-profit and provided a workaround to insurance. The hesitance and ambivalences felt by clinicians and stakeholders was socially organized (Benjamin 2011; 2013) and navigated through moral and normative assessments of how economics ought to be embedded in cancer research and treatment. Clinicians and stakeholders drew connections between genomic platforms, the promise of a “local” biopharmaceutical industry and research enterprise, and the moral imperative to address systemic rural cancer care disparities. This “localization” legitimated investments in cancer genomics and relaxed the moral tensions that clinicians and stakeholders faced in attempting to “do good” within what they saw as deeply flawed and unequal healthcare delivery systems. The contradictions and moral ambiguities of US bio-economies of cancer thus generated a “mix of acceptance and counterdiscourse” that created space for critiquing processes of biomedicalization and current biomedical economies while nonetheless maintaining their momentum (Whitmarsh 2008, 9; A. E. Clarke *et al*. 2010). Indeed, MCGI was considered successful precisely because oncologists from nearly every practice in the state ultimately participated.

On the one hand these findings speak to how frustrating and ambivalent feelings about the economies of cancer genomics ultimately maintained the momentum of ongoing biomedicalization. On the other hand, these findings point to the value of taking the feelings of biomedical practitioners seriously. The affective expressions of moral tension and ethical excitement we analyse provide a slim wedge that may open socially reflexive questions about how “responsible understandings of value in biomedicine and healthcare ought to be” (Datta Burton *et al*. 2021, 2). The questions, critiques and hesitance that clinicians and stakeholders expressed about commercial ventures, profit motives and business models may point toward news ways of reflecting upon and governing value structures in bioresearch and healthcare delivery. Many of the conflicts and tensions clinicians and stakeholders identified did not dwell within the actions of individual investigators or institutions. In other words, while we certainly heard concerns about potential “proprietary interests,” many of the sources of conflicts stakeholders described feeling could not be easily personalized or individualized. Rather, the uneasy contradictions felt by clinicians and stakeholders point to questions about how to construct locally “good” cancer genomics within flawed and stratified systems that they characterized as valuing the wrong things. Precision oncology simultaneously represented the spiraling costs of cancer tests and treatment, as well as a strategy for strengthening the biomedical workforce and contributing to economic development in the region; “health” operated simultaneously as a cost that must be controlled and a market to be grown (Dumit 2012). Conflict of interest disclosures and physician payment transparency policies focus on financial incentives that particular individuals may be subject to; there are consequently few possibilities for collectively acting on or governing the complex chain of healthcare drivers or paradoxical institutional incentives of US biomedicine (Dumit 2012; Kaufman 2015). What might it mean, however, to reconsider individualized “conflicts of interest” in terms of the underlying values structures and infrastructures that ultimately generate these conflictual tensions and shape how biomedical actors respond within “orders of desirability” or structures of feeling? Might a deeper consideration of how the anchoring infrastructures that animate conflicted values in biomedical practice open up new ways of governing, enacting and interrogating “good” science and care in the post-genomic era? To raise up Kaufman’s critical question, might it help us ask: “What values do we want to govern [the biomedical] enterprise?” (Kaufman 2015).

## Figures and Tables

**Table 1.** Composition of study participants.

Professional Role	*N* =
MCGI Clinicians	20
Health System/Administration	5
Philanthropy	2
Pathology	3
Biomedical Research/Genetics	4
**Total:**	**n**=**34**

## Footnotes

1. Few needed to be convinced that “science and ethics interact” in the controversial domain of stem cell research in California. Thompson contrasts this always already “ethicized” science of stem cell research with a “conventional view of the ethics of science made up a of a professional code of conduct (don’t fake your results, don’t steal my reagent) and possible downstream ethical, legal or social implications (after the science is over, are the results used for good or ill?)” (Thompson 2013, 27).
